# Rare finding of a symptomatic epidermoid cyst of the diaphragm – a case report

**DOI:** 10.1093/jscr/rjae111

**Published:** 2024-03-06

**Authors:** Fraser Welsh, Frank Weilert, Bernd Grunewald, Laxmi Lanka, Sudha Patil

**Affiliations:** Department of General Surgery, Te Whatu Ora Waikato, 183 Pembroke Street Hamilton, Waikato 3204, New Zealand; Deparment of Gastroenterology, Te Whatu Ora Waikato, 183 Pembroke Street Hamilton, Waikato 3204, New Zealand; Department of General Surgery, Te Whatu Ora Waikato, 183 Pembroke Street Hamilton, Waikato 3204, New Zealand; Department of Radiology, I-MED Radiology Network, 11 Thackeray Street Hamilton 3204, New Zealand; Department of Pathology, Pathlab Waikato, 58 Tristram Street Hamilton 3204, New Zealand

**Keywords:** epidermoid cyst, diaphragm, case report

## Abstract

Cystic lesions of the diaphragm are rare and accordingly present a diagnostic challenge. Specific radiological features with which to clinch a diagnosis may be elusive. Herein we present the case of a patient who presented with symptoms attributable to a cyst in the left upper abdomen, irritating the diaphragm. Surgery was considered appropriate for diagnostic and symptomatic purposes. Final histology demonstrated an epidermoid cyst. Resolution of symptoms was reported after surgery. Diaphragmatic epidermoid cysts appear to be a rare entity with only three prior cases reported in the literature. Given the rarity of this lesion and the lack of unique features by which they can be characterized, accurately diagnosing epidermoid cysts of the diaphragm is likely to remain difficult without surgery, although they are presumed to have a benign behaviour.

## Introduction

This account describes a patient who presented to an urban hospital with symptoms attributed to the finding of a cystic lesion irritating the diaphragm. Surgery was offered to remove the cyst, with resolution of the symptoms. Histology demonstrated the lesion to be an epidermoid cyst. Description of diaphragmatic epidermoid cysts is exceedingly rare hence this benign entity may not be considered in a radiological differential diagnosis. Given the lack of imaging features specific to epidermoid cysts, surgical pathology may be the only practical means of a definitive diagnosis. The following work has been reported in line with SCARE criteria [1].

## Case report

A 41-year-old female self-presented via the Emergency Department with left shoulder pain that worsened with breathing. There was no history of injury nor was there any other specific clinical examination finding.

Her background included ulcerative colitis (in remission for 7 years on mesalazine and mercaptopurine), pyoderma gangrenosum and a Covid-19 infection 6 weeks prior. Blood tests were normal including liver function tests, lipase, and troponins. Electrocardiogram and chest x-ray were normal. A diagnosis of musculoskeletal pain was made and treated with analgesics.

**Figure 1:**
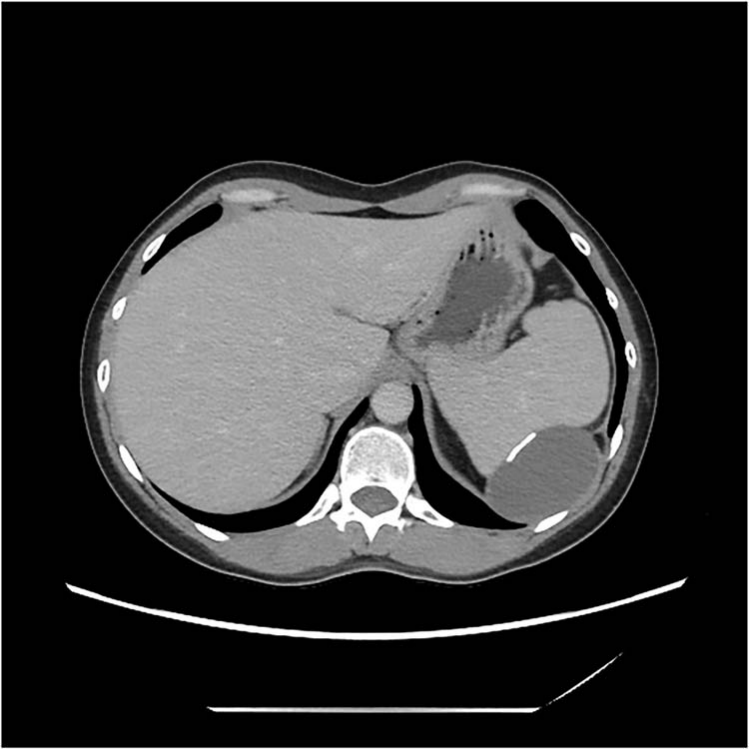
CT image demonstrating cyst adjacent to spleen and diaphgram with calcified walls.

A computerised tomography (CT) scan was organized thereafter, demonstrating a cystic lesion between spleen and diaphragm. The lobulated cyst was 7.3 × 6.7 × 4.2 cm. The lesion had a slightly thickened wall with scattered coarse calcifications. The cyst indented and appeared inseparable from the spleen. No peritoneal disease was evident (Fig. 1).

After discussion in a multidisciplinary forum, surgical excision was recommended. Due to concern over lack of clear planes between diaphragm, cyst and spleen with possible need for splenectomy, vaccinations for encapsulated organisms were given up-front. Open, rather than minimally invasive surgery was preferred, anticipating that surgery may be difficult.

**Figure 2:**
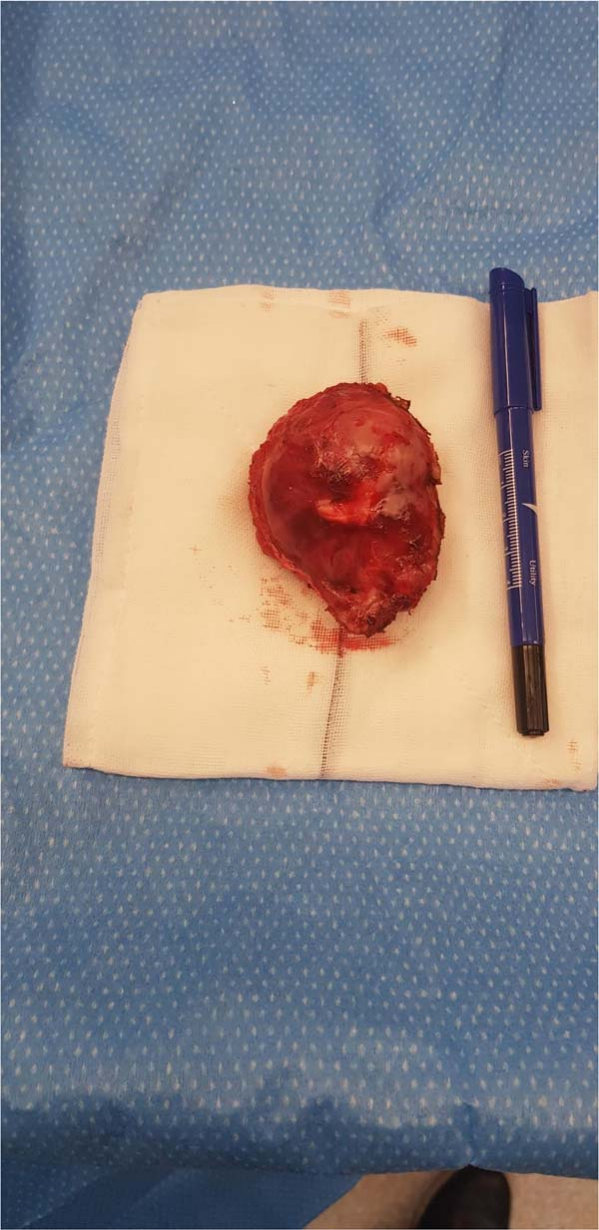
Surgical specimen – cyst excised with disc of diaphragm.

At surgery, the abdomen was accessed via a left subcostal incision. The spleen was found to be separate from the cyst which was attached to the diaphragm. The cyst was excised with a disc of diaphragm from the left costophrenic recess (Fig. 2). The defect was closed with interrupted vicryl sutures, after expelling air from the chest into an underwater sealed drain. The abdomen was closed, and the patient recovered well. At two-month follow up the pre-operative symptom of shoulder tip pain remained resolved.

**Figure 3:**
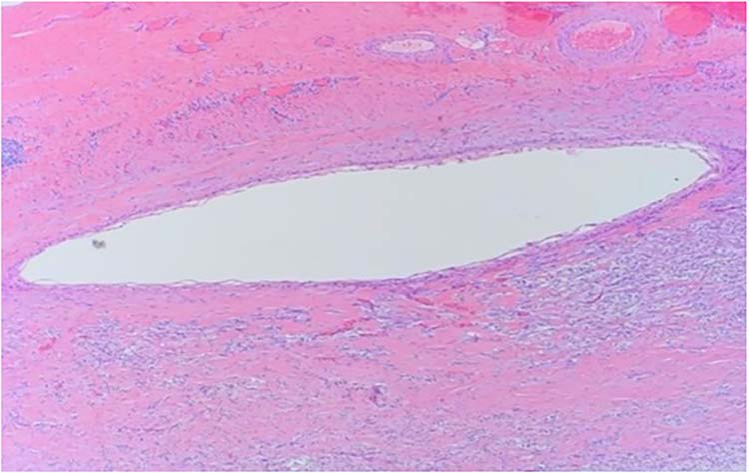
Close up of squamous epithelium.

**Figure 4:**
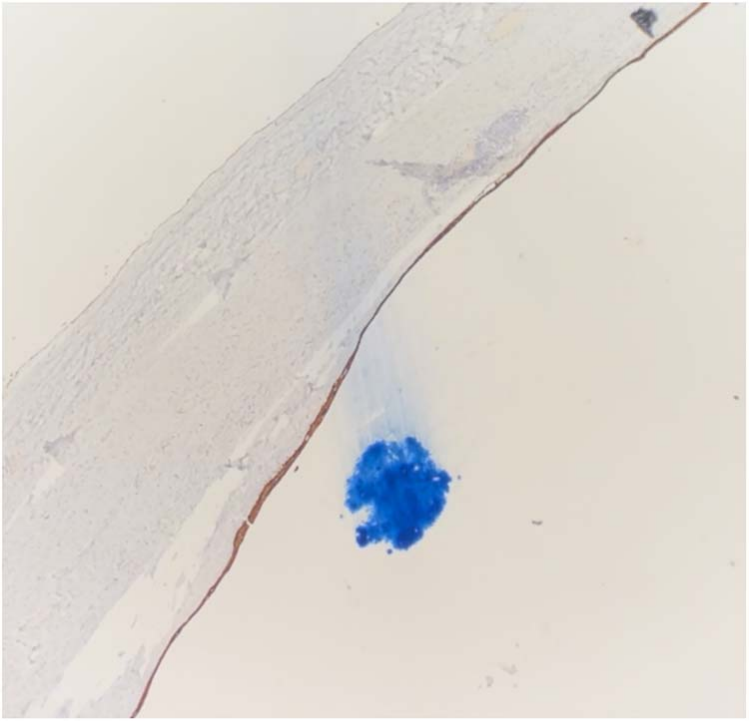
Squamous epithelium demonstrating CK staining.

**Figure 5:**
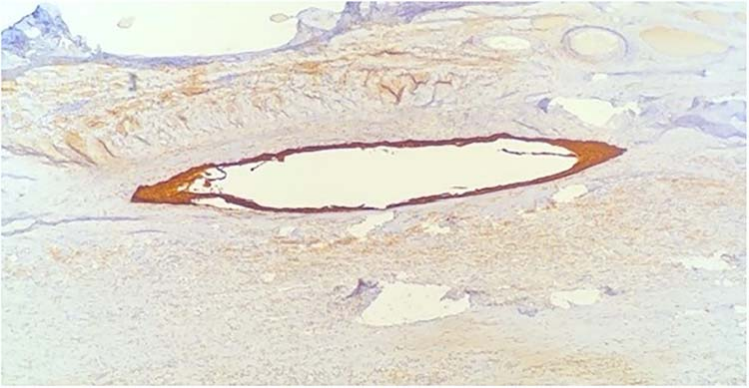
CK5 positive staining.

Histology demonstrated a cystic mass 75 × 33 × 36 mm. The cyst wall was covered with peritoneum showing focal fibrous adhesions and congestion. On one side of the cyst was a white nodular calcified area with associated congestion. Microscopy showed a fibrotic cyst, lined with inflammatory infiltrate. The cyst contained amorphous eosinophilic material. The cyst wall showed focal calcifications, fibrosis and predominantly denuded lining epithelium. Where preserved, the wall was lined by squamous epithelium (Fig. 3) highlighted by CK5 and CK immunostains (Figs 4 and 5). Mesothelial markers Calretinin and WT1 were negative. There was no evidence of atypia, dysplasia or invasive malignancy. It was concluded that this was an inflamed epidermoid cyst.

## Discussion

Cystic lesions between the spleen and diaphragm are rare but may be detected by cross sectional imaging. Features in the clinical history or imaging may help characterize a diaphragmatic cyst to guide management, but in this case the diagnosis of an epidermoid cyst could not be made until after surgery. Epidermoid cysts of the diaphragm are very rare with three prior cases reported in the literature [2].

Epidermoid cysts are benign tumours otherwise known as epithelial or epidermal cysts [3]. Non-cutaneous epidermoid cysts are uncommon but can be found anywhere in the body. Splenic epidermoid cysts are well-reported but diaphragmatic lesions seem particularly uncommon.

Generally, epidermoid cysts appear as ovoid structures with a non-infiltrating rim on imaging studies [4]. In locations where epidermoid cysts are unusual these features are non-specific. While radiological descriptions peculiar to diaphragmatic epidermoid cysts are scarce, splenic epidermoid cysts are usually unilocular, well demarcated and thin-walled, with low-density contents on CT. As was noted in this cyst, wall calcifications may occur. Radiological biopsies are usually avoided due to risk of complications [5].

Differentiating epidermoid cysts from other more recognized peri-diaphragmatic cysts can be difficult. Although calcifications were noted in this case, the relatively thin walls and absent history of trauma made a traumatic pseudocyst unlikely [6]. Diaphragmatic mesothelial cysts are benign lesions derived from coelomic remnants. In contrast to this case, they typically present in the paediatric population. Right sided lesions are more commonly described. Splitting of diaphragmatic fibres around the cyst is characteristic of a mesothelial cyst on targeted ultrasound [7] but this was not identified as a feature on CT. Unlike this case, congenital bronchogenic cysts are more usually discovered in the posterior mediastinum or lung parenchyma [8]. Echinococcal cysts can occur in the diaphragm [9] or spleen [10]. Daughter cysts and the presence of ‘hydatid sand’ may be pathognomic on imaging studies. Again, these features were absent.

Histological features of epidermoid cysts include squamous epithelium and positive staining for CK [2], as was present in this case. Unlike a dermoid cyst, skin adnexae like hair follicles and sebaceous glands were absent.

The pathogenesis of diaphragmatic epidermoid cysts is uncertain. Splenic epidermoid cysts have been proposed to arise from squamous metaplasia of mesothelial cysts [11]. Little is known about the natural history of epidermoid cysts of diaphragm. On histological grounds and by analogy with cutaneous epidermoid cysts, they are presumed to be benign. Raised CA19-9 levels which returned to normal levels after resection of benign epidermoid cysts has been described [2, 12], suggesting that this serum marker may not help differentiate malignant from benign cysts.

Laparoscopic or open surgery can be considered for cystic lesions in this region if symptomatic or if concerned about a non-benign pathology on radiological or clinical grounds [13]. It has been suggested that cysts >5 cm in size may be at risk of rupture [2]. This case posed a diagnostic challenge, given the lack of specific radiological or clinical features. The final diagnosis could only be made after surgery. Diaphragmatic epidermoid cyst has rarely been reported and accordingly may be overlooked as a potential cause for a cystic lesion in the diaphragm.
